# Development assistance for human resources for health, 1990–2020

**DOI:** 10.1186/s12960-022-00744-x

**Published:** 2022-06-10

**Authors:** Angela E. Micah, Juan Solorio, Hayley Stutzman, Yingxi Zhao, Golsum Tsakalos, Joseph L. Dieleman

**Affiliations:** 1 Institute for Health Metrics and Evaluation, Population Health Building/Hans Rosling Center, 3980 15th Ave. NE, Seattle, WA 98195 USA; 2 Department of Health Metrics Sciences, University of Washington, Seattle, WA 98195 USA; 3 Nuffield Department of Medicine, University of Oxford, Oxford, OX3 0JE UK

**Keywords:** Development assistance for human resources for health, Global Strategy for Human Resources for Health, Donor support, Health workforce

## Abstract

**Background:**

Investing in the health workforce is key to achieving the health-related Sustainable Development Goals. However, achieving these Goals requires addressing a projected global shortage of 18 million health workers (mostly in low- and middle-income countries). Within that context, in 2016, the World Health Assembly adopted the WHO Global Strategy on Human Resources for Health: Workforce 2030. In the Strategy, the role of official development assistance to support the health workforce is an area of interest. The objective of this study is to examine progress on implementing the Global Strategy by updating previous analyses that estimated and examined official development assistance targeted towards human resources for health.

**Methods:**

We leveraged data from IHME’s Development Assistance for Health database, COVID development assistance database and the OECD’s Creditor Reporting System online database. We utilized an updated keyword list to identify the relevant human resources for health-related activities from the project databases. When possible, we also estimated the fraction of human resources for health projects that considered and/or focused on gender as a key factor. We described trends, examined changes in the availability of human resources for health-related development assistance since the adoption of the Global Strategy and compared disease burden and availability of donor resources.

**Results:**

Since 2016, development assistance for human resources for health has increased with a slight dip in 2019. In 2020, fueled by the onset of the COVID-19 pandemic, it reached an all-time high of $4.1 billion, more than double its value in 2016 and a 116.5% increase over 2019. The highest share (42.4%) of support for human resources for health-related activities has been directed towards training. Since the adoption of the Global Strategy, donor resources for health workforce-related activities have on average increased by 13.3% compared to 16.0% from 2000 through 2015. For 47 countries identified by the WHO as having severe workforce shortages, the availability of donor resources remains modest.

**Conclusions:**

Since 2016, donor support for health workforce-related activities has increased. However, there are lingering concerns related to the short-term nature of activities that donor funding supports and its viability for creating sustainable health systems.

**Supplementary Information:**

The online version contains supplementary material available at 10.1186/s12960-022-00744-x.

## Background

Adequate numbers of health workers who are equipped with the knowledge, skills, and tools to deliver quality health care is essential for providing effective coverage that can deliver on the health-related Sustainable Development Goals. In 2016, the World Health Assembly adopted the Global Strategy on Human Resources for Health (GSHRH): Workforce 2030 [1]. A key challenge the strategy aimed to address was a projected need for an additional 18 million health workers mostly in low- and middle-income countries [2, 3]. Addressing this challenge is critical to achieving the health-related Sustainable Development Goals. Given this context, the Global Strategy aims to improve the health, social and economic outcomes of all by ensuring universal availability, accessibility, acceptability and quality health workforce through appropriate investments and policy implementation [1]. To make the strategy actionable, it provided relevant policy options for key stakeholders. And to monitor its progress, it included thirteen milestones, some to be achieved by 2020 and others by 2030.

One of the areas of interest in the GSHRH is the role of official development assistance to support the health workforce. Specifically, milestone four of the GSHRH focused on “*increasing synergies in official development assistance for education, employment, gender and health, in support of national health employment and economic growth priorities*” by 2030 [1]. The inclusion of this milestone highlights the substantial investment in terms of resources and multi-sectoral collaboration needed in order to be able to address the estimated deficit in health workforce and to deploy the appropriate level and skill mix of health workers for effective coverage. Beyond national government resources, it points to the need for external support from development partners and a greater coordination of that support.

In low- and middle-income countries, there is limited information on how much governments and development partners are providing in support of health workers. While anecdotal evidence suggests that in some low- and middle-income countries a high percentage of government funding of the health sector go towards the salaries of health workers [4, 5], data on external support for health workforce-related activities are even more limited. Academic and grey literature provides examples of donor governments providing various forms of health workforce-related support. This is especially so, after initial efforts showed that the achievement of target outcomes set by development agencies such as the Global Fund for HIV/AIDS, Tuberculosis and Malaria (GFATM) and the President’s Emergency Plan for Aids Relief (PEPFAR) were constrained by the shortage of health workers [6, 7]. This realization galvanized donor investment in the health workforce-related activities in countries of high priority and relevance [8–11]. A previous study estimated that only 4% of development assistance for health has been targeted towards human resources for health, when the Global Strategy was set in 2016 [12]. Given the magnitude of the need, development assistance will be essential to boost what national governments are able to mobilize and for resourcing areas where gaps remain due to severely constrained budgets in low-income countries.

Tracking development assistance for activities related to human resources for health is important for monitoring progress, identifying outstanding gaps, and planning for the achievement of milestone four of the GSHRH. The objective of this study is to assess progress by updating previous analyses that estimated and examined development assistance targeted towards human resources for health. In addition to reporting the source, channel and recipient of these funds, the estimates were categorized by type of investment and how focused they were on gender disparities.

## Methods

We leveraged data from the Institute for Health Metrics and Evaluation’s (IHME) Development Assistance for Health database (DAH), COVID development assistance database and the Organization for Economic Cooperation and Development’s Creditor Reporting System (CRS) online database [13–15]. We relied on the CRS for project-level data for the following international development agencies: Development Assistance Committee member’s bilateral agencies, Non-governmental organizations, European Commission, Global Fund, WHO and the UN agencies. For the Bill and Melinda Gates Foundation, World Bank and the regional development banks—African Development Bank, Asian Development Bank and Inter-American Development Bank we leverage data utilized in the IHME Development Assistance for Health database. We obtained data from Candid on other US foundations [16]. IHME’s databases leverage project descriptions from online databases of international development agencies and annual financial statements to generate estimates of development assistance for health.

For the following development agencies included in the analyses—Global Fund, Gavi and the UN agencies, because project-level data were not available in the IHME database, we utilized the CRS data on these disbursing agencies to calculate the proportion of human resources for health-related activities reported. We then multiplied this proportion with the total development assistance for health for these disbursing entities reported in the IHME database. We therefore did not have information on the source of these funds and designated these as unallocable sources.

The methods used to generate the estimates for development assistance for health workforce-related activities are documented elsewhere [12]. However, we made several modifications to accommodate improvements in the underlying data. Furthermore, we generated estimates for the most recent year (2020) for which project-level data are yet to become available in the CRS online database in order to examine changes brought on by the COVID-19 pandemic. Improvements in the underlying database includes the addition of China in the updated development assistance for health database [17]. This extension of the database captures a snapshot of development assistance flows among low- and middle-income countries. The data included on China is not available at the project level and therefore we are unable to further disaggregate into the types of human resources for health-related support although we have an estimate of the overall support provided.

Additionally, in this dataset, we utilized an updated list of keywords when searching project titles and descriptions to isolate the relevant human resources for health-related activities from the project databases. We added keywords to isolate health worker information systems as a type of human resources for health activity. Additional file 1: Table S1 in the Appendix presents the list of keywords used for isolating the types of human resources for health activities. As in the previous analyses, the types of activities highlighted included staffing, training, education, infrastructure, administration and policy, other with new additions for health workforce information systems. Staffing characterizes the hiring of additional personnel or consultants to increase the available labor supply. Training covers pre-service and in-service training activities such as on-the-job training and internships. Education captures sponsorship opportunities that allow health workers to complete pre-service or post-graduation education. Infrastructure covers activities such as the provision of equipment for health facilities and the building of health institutions. Administration and policy activities focus on building leadership, management and policy development skills among health workers. Health workforce information systems characterize activities that develop information systems that collect and disseminate data on the health workforce in a country [18].

Where the data allowed, we also accounted for gender equity across projects. These included projects with activities that enable or promote an equitable work environment for men and women [19]. We did not use the keyword approach to isolate the projects related to gender, instead we utilized information provided in the CRS dataset in the gender marker variable [20]. This is a variable in the dataset that donors use to specify the extent to which a project had activities that supported gender equity. Projects could be assigned three categories: (i) not targeted, (ii) significant, (iii) principal. Projects categorized as not targeted had no gender equality-related activities, whereas projects categorized as significant had some and those categorized as principal where primarily focused on gender issues. We designated projects that were marked as significant or principal as gender related. Assuming a project designated as principal in the gender marker had no other relevant keywords then we assigned the entire value of the project to the gender category. However, if there were other types of keywords tagged, we proportioned it to assign the same value to gender as the other highest computed types. No estimates of human resources for health gender equity activities were made for 2020 because the project-level data from the CRS were available only till 2019.

In order to include estimates for the most recent year, 2020, we leveraged the human resources for health total estimate from the IHME DAH and COVID databases. The COVID database leveraged additional data sources—United Nations Office for the Coordination of Humanitarian Affairs and the International Aid Transparency Initiative as well as correspondence data—to generate estimates of donor funding for the health-related COVID-19 response [21, 22]. Together with the DAH database, we can estimate the total value of donor contributions for 2020. From the DAH database, we pulled the 2020 total DAH estimates and used a 3-year weighted average of the by type fractions to disaggregate this total into by type allocations. To isolate human resources for health-related activities among the COVID-related projects, we ran the keywords search utilized previously on the project-level development assistance for COVID database. This process flagged the relevant projects for inclusion.

We described trends in funding contributed by donors for human resources for health-related activities, used growth rate regressions to determine average growth rate pre and post the GSHRH and compared disease burden and availability of donor funding for human resources for health. The latter analysis highlights the 47 support and safeguard countries identified by the WHO as having the most pressing health workforce-related needs. All data are reported in 2020 US dollars. R version 3.6.1 was used for the analyses.

## Results

**Fig. 1:**
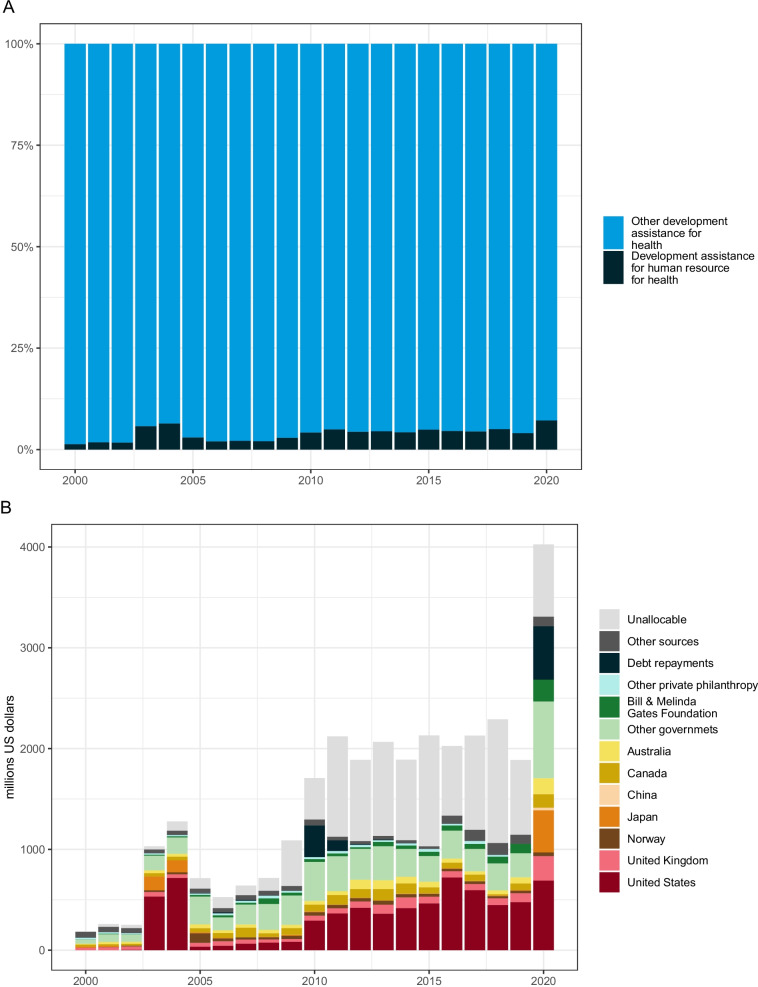
Development assistance for human resources for health; 2000–2020. **A** Share of development assistance for human resources for health of total development assistance for health. **B** Development assistance for human resources for health by source of funding; billions of 2020 USD. “Other” captures development assistance for health for which we have source information, but which is not identified as originating within any of the sources listed. Health assistance for which we have no source information is designated as “Unallocable”. The 2020 bar presents development assistance for human resources for health and COVID-19 with source information originating from the data

Development assistance for health workforce has increased over time especially with the onset of COVID-19, but it still only represents a small share of total development assistance for health. Figure 1 shows how development assistance contributed towards human resources for health-related activities has evolved from 2000 through 2020. Overall, the development assistance for human resources for health contributed, in absolute value and as a share of total development assistance for health, has increased over time. While there was a decrease in funding from 2018 ($2.29 billion) to 2019 ($1.89 billion), because some key projects ended, in 2020, it reached an all-time high of $4.09 billion, more than double its value in 2016 when the Global Strategy was adopted and a 116.5% increase over its 2019 value. Furthermore, as a proportion of total development assistance for health, contributions towards human resources for health-related activities was at least 5% each year since 2016 (panel A) The main sources of development assistance towards human resources for health-related activities have remained consistent across time, with the United States contributing the most ($691.8 million) and United Kingdom and Canada contributing $241.8 million and $134.7 million, respectively, for 2020. Also of note is China’s contribution of $25.1 million (panel B). Some sources have peculiar disbursement patterns over time. For the United States, the data suggest that project descriptions might have been missing or the included description was inadequate to be flagged as relevant by the keywords used in the study. For Japan, the patterns observed suggest that some larger multi-year projects provided most of their commitment in the final years of the project while subsequently spreading the disbursement more evenly for later projects.

**Fig. 2:**
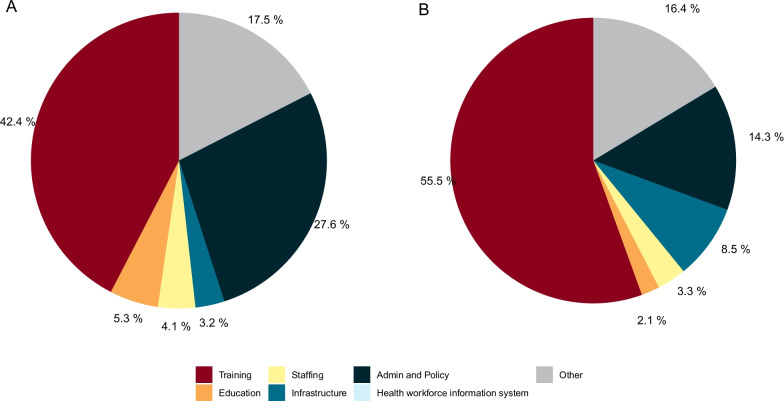
Development assistance for human resources for health by type; 2016–2020. **A** Share of development assistance for human resources for health by type of focus area, 2016–2019. Note: Shares were calculated through the weighted average of the most recent three years of non-COVID data (2016–2019). **B** Share of development assistance for human resources for health by type of focus area, COVID-19. Limited donor supported activities related to health workforce information systems were found through our search methodology, however the label remains in the data

**Fig. 3:**
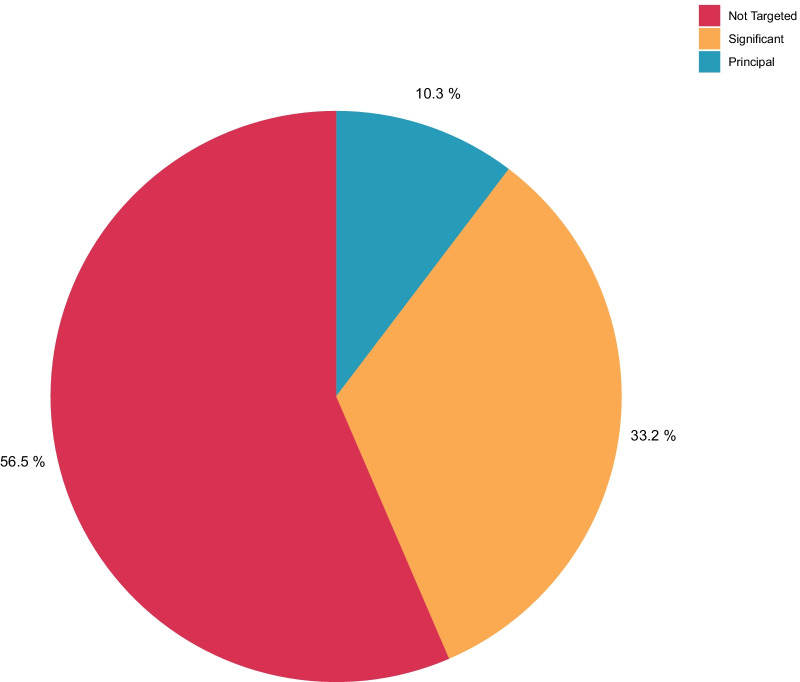
Development assistance for human resources for health by gender equality focus assignation; 2016–2019. Note: Weighted average of most recent 3 years of non-COVID data (2016–2019). “Not targeted” refers to projects that had no gender equality-related activities, whereas projects categorized as “Significant” had some gender equality-related activities and those categorized as “Principal” where primarily focused on gender issues. Assuming a project designated as principal in the gender marker had no other relevant keywords then we assigned the entire value of the project to the gender category. However, if there were other types of keywords tagged, “Significant” projects were proportioned it to assign the same value to gender as the other highest computed types. “Principal” projects were a higher amount value as the other highest computed types

The main program area of support is training. Figure 2 presents the types of human resources for health-related activities that have been funded since 2016 when the Global Strategy for Health Workforce was adopted. Panel A shows the distribution of funding between 2016 through 2019 while panel B highlights the types of human resources for health activities that were funded during the COVID-19 pandemic in 2020. Between 2016 and 2019, the largest fraction (42.4%) of support for human resources for health-related activities have been directed towards training activities. Administration and policy-related activities made up the next largest share, with a little over a quarter of resources (27.6%) directed towards activities that support the creation of policies and management plans for the health workforce. Limited donor supported activities related to health workforce information systems were found. While the distribution pattern of supported activities was similar, in 2020, a relatively substantial (more than half, 55.5%) was targeted towards training. Furthermore, infrastructure-related activities seem to have received more than double (8.5%) the amount that had historically been targeted towards such activities (Panel b). Of all projects that were examined for gender components, 27.5% involved funded activities related to gender (Fig. 3).

**Fig. 4:**
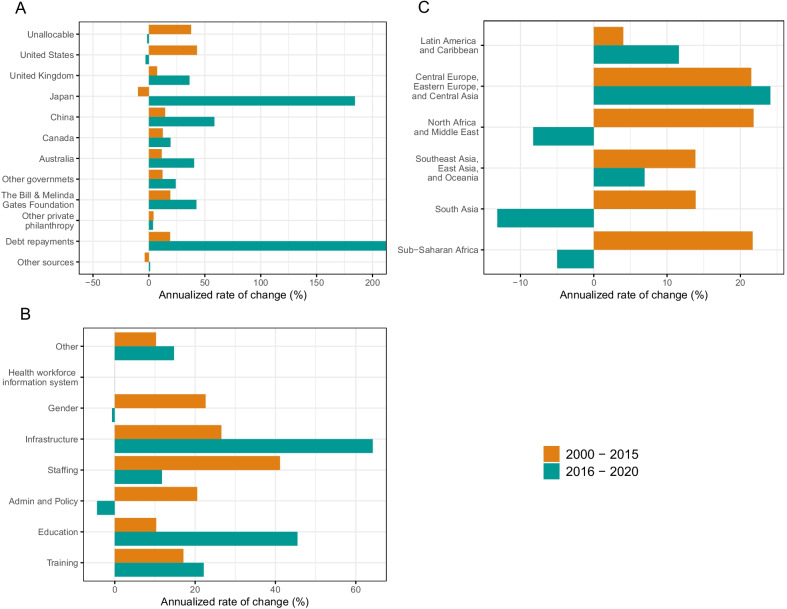
Annualized rate of change in development assistance for human resources for health. **A** Annualized rate of change in development assistance for human resources for health disbursed by source. **B** Annualized rate of change in development assistance for human resources for health disbursed by type of human resources for health. **C** Annualized rate of change in development assistance for human resources for health disbursed by region

**Fig. 5:**
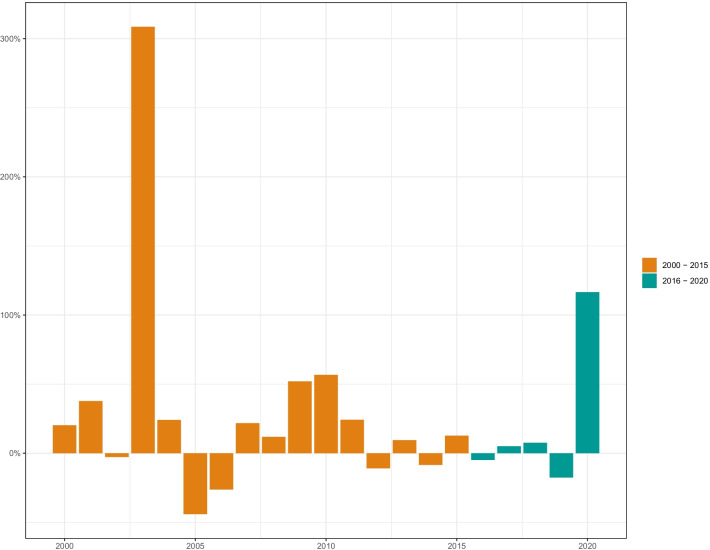
Year on year percent difference in development assistance for human resources for health; 2000–2020. Growth in donor funding for human resources for health activities prior to the adoption of the Global Strategy (2000–2015) and the period after (2016–2020)

The growth in available resources to support health workforce-related activities was higher during the pre GSHRH era. While the United States remains the main source of development assistance for human resources for health, the growth in contributions from Japan, China and Bill and Melinda Gates Foundation have recently also been significant. Across targeted regions, the growth is donor support for human resources for health activities has been nuanced with decreases in the recent past in some regions. Figure 4 highlights the growth in donor funding for human resources for health activities prior to the adoption of the GSHRH and after. In panel A, we observe the changes in the growth rates in donor support across the two time periods by the source of funding. Overall, growth rates in the period 2000–2015 were higher (16% compared to 13%) than in the period 2016–2020. Between 2000 and 2015, sources of donor support for human resources for health activities with the highest growth rate were United States (43%) and Norway (27%) whereas since 2016 we observe Japan (184%) and China (59%) as the donors with the highest growth rates. Panel B showcases the growth rates in donor support across specific types of human resources for health pre and post GSHRH. Here, we observe high growth rates in staffing (41%) and infrastructure (27%) pre–Global Strategy. While post Global Strategy, infrastructure (64%) and education (45%) were the activities for which we observe substantive increases in targeted funding. Lastly, panel C presents the comparison of growth rates pre and post Global Strategy by Global Burden of Disease super region. Prior to 2016, we observe growth in human resources for health-related development assistance across all regions. On the contrary, post 2016, negative growth is observed in all super-regions except Latin America and the Caribbean. The rate of growth in donor funding was highest in North Africa and Middle East (24%), sub-Saharan Africa (22%) and Central Europe, Eastern Europe, and Central Asia (22%) pre 2016 and the largest decreases in growth rate of funding are in Central Europe, Eastern Europe, and Central Asia (24%) and Latin America and Caribbean (12%) post 2016. The average year-on-year difference was also higher pre GSHRH (30%) compared to post GSHRH (21%) (Fig. 5).

**Fig. 6:**
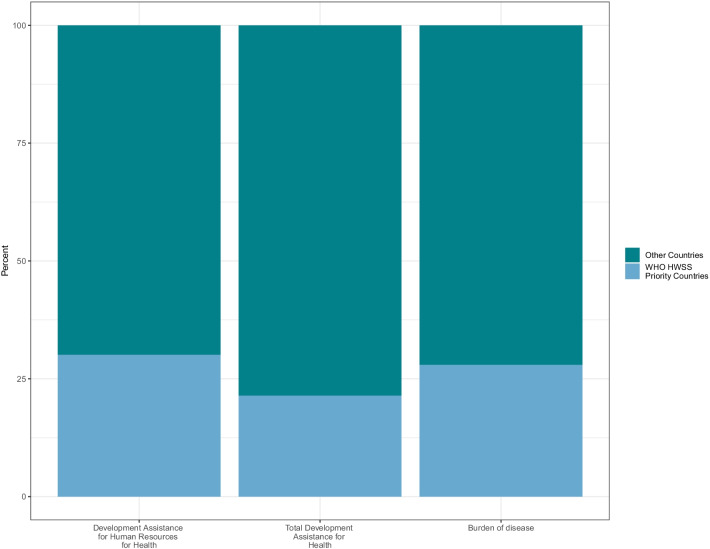
Needs-based health worker shortages and availability of development assistance by WHO priority countries. Note: WHO Health Workforce Support and Safeguards (HWSS) Countries fall into the WHO regions, comprises 47 countries, that face the most pressing health workforce challenges related to UHC: Africa: Angola, Benin, Burkina Faso, Burundi, Cameroon, Central African Republic, Chad, Congo, Cote d Ivoire, Democratic Republic of the Congo, Equatorial Guinea, Eritrea, Ethiopia, Gabon, Gambia, Ghana, Guinea, Guinea-Bissau, Lesotho, Liberia, Madagascar, Malawi, Mauritania, Mozambique, Niger, Nigeria, Senegal, Sierra Leone, South Sudan, Togo, Uganda, United Republic of Tanzania. Americas: Haiti. Eastern Mediterranean: Afghanistan, Djibouti, Pakistan, Somalia, Sudan, Yemen. South-East Asia: Bangladesh, Nepal. Western Pacific: Kiribati, Micronesia (Fed States of), Papua New Guinea, Solomon Islands, Vanuatu

Figure 6 compares overall development assistance for health, development assistance for human resources for health and the disease burden. We highlight the 47 support safeguard countries identified by the WHO as having the most pressing Universal Health Coverage health workforce-related needs. For these countries, the unadjusted share of human resources for health-related funding (30%) is on par with their disease burden (28%), although the share of overall development assistance for health targeted towards these countries is lower (17%).

## Discussion

This study leveraged updated data to provide new estimates of development assistance for human resources for health. These new estimates are then used to examine donor funding flows for health workforce-related activities since 2016 when the Global Strategy on Human Resources for Health: Workforce 2030 was adopted by WHO member states. A key finding is that over time donor support for health workforce activities has risen with a distinct increase in funding for 2020 due to the COVID-19 pandemic. We estimated that since the adoption of the Global Strategy in 2016, $12.4 billion has been contributed towards human resources for health-related activities by development partners. Furthermore, the types of activities supported, the main sources and recipients of donor funding for human resources for health have changed marginally over time. Training remains the primary type of human resource-related activity supported by donors.

Our estimate of $4 billion (7%) of overall development assistance for health in 2020 being targeted towards human resources for health-related activities are similar in value (but much lower in percent) to the estimate provided by Catherine Michaud (2004) in her estimation for the Joint Learning Initiative. Per the Michaud analyses, about 40 percent of development assistance for health is targeted towards human resources for health activities [23]. The difference is to be expected since different methodologies were used although both analyses utilized data from the OECD CRS online database. Michaud relied primarily on OECD purpose-code (medical education) assignment while we utilized keywords to isolate relevant projects. Additionally, these new estimates are higher than previous estimates generated by this study team. The sources of increase include updated data that capture additional sources of funding such as China and revised keywords list that identify new types of health workforce-related activities.

While development assistance for human resources for health activities has continued to increase in aggregate post adoption of the Global Strategy, there are two important issues to note. First, the share of total DAH that is targeted towards human resources for health remains small. This is especially so when compared to the suggested 40 percent used by Michaud based on a detailed examination of donor reports, program expenditures and national health accounts in 2004. Given the centrality of human resources for health in achieving key global goals this finding suggests that more resource mobilization is appropriate and that there may be the fiscal space for it as well especially based on how the pandemic has enabled a doubling of available funds for health worker support.

Second, the bulk of donor support for health workforce-related activities remains focused on short-term training activities. This orientation was magnified in 2020, with the onset of the pandemic. While the drivers of this donor inclination are known, ultimately this position conflicts with attempts to foster sustainable health systems. This is because while such activities help alleviate immediate competency needs it fails to address the underlying causes of health worker shortages such as the high cost of producing and employing health workers, a growing and aging population, and internal and international migration [24]. In order to promote sustainable health systems, more support for activities such as those that facilitate management of health workers or promotes their retention is critical. Milestone four for monitoring the Global Strategy promotes synergizing donor efforts. This challenge with the short-term nature of human resources for health supported projects is one area that presents a unique opportunity for synergy among donors in order to promote the goal of ensuring sustainable health systems. Similarly, efforts by donors and national governments to ensure that the support provided is in line with country capacity and priorities will promote a more efficient use of limited resources.

Additionally, Stenberg and colleagues estimate that an additional $371 billion will be needed each year for 67 low- and middle-income countries to reach the health-related targets by 2030. Seventy-five percent ($278 billion) of this additional money needed to go towards health system strengthening, which is inclusive of health workforce strengthening, infrastructure for service delivery and health information systems. For health worker strengthening activities, between $92 and $150 billion (depending on scenario) in additional yearly funding is estimated to be needed [37]. The magnitude of this estimate highlights that dramatic increases in both donor and government resources for human resources for health are needed if the Sustainable Development Goals are to be met.

The COVID-19 pandemic presents peculiar challenges for health workers. The data from this study suggest that like overall development assistance for health, development assistance for human resources for health also saw a dramatic increase in 2020. However, most of this additional support seems to be targeted towards training health professionals as well as community health workers and volunteers in order to facilitate the delivery of care during the pandemic. While this seems justified as a critical need in the moment, the approach still perpetuates the emphasis on short-term activities and does not support the desire to promote sustainable health systems.

The issue of gender equity in human resources for health is a complex one. The main area of concern is that there is a disproportionate representation of women in service-oriented lower ranked health care roles than more senior managerial roles [25–27]. Donor interest and hence the availability of resources targeted towards this issue has increased in the recent past. Vera and colleagues estimate that $798 million, 66% of overall development assistance funding in 2017 was targeted towards this area [28]. Separately, the OECD also reports that $4.4 billion (51%) of development assistance for health is targeted towards this area on average annually [29]. Where the data allowed us to examine gender equity, the estimates in this study suggest that on average 28% of development assistance for human resources for health between 2016 through 2019 had gender components. An example of such projects is one that focused on providing scholarships for health professionals to pursue their Graduate education (Masters level) at an African or US university with women’s organization as the local implementing partner. Yet still, there remain strong sentiments that more resources need to be dedicated to this issue given the many political commitments that have been made denouncing the status quo that women make up two-thirds of the health workforce especially the majority of nursing and midwifery workforce and yet still face severe inequities [30, 31]. Investment in gender-transformative initiatives are needed to address inequity in training and employment opportunities and terms (especially huge gender pay gap), workplace safety as well as career advancement and serving in leadership positions [30].

It should also be noted that development assistance for human resources for health is only one pillar of global human resources for health spending. Country governments also heavily invest in the health workforce through providing workforce training, salary and remuneration among other activities, which usually take up one-third of total government expenditure on health [1]. While a lot of pre-service and in-service training are sponsored by government or paid by health workers themselves, in the last decade there has been a huge increase in private sector investment in medical education for example bilateral education cooperation between private medical institutions in high-income or middle-income countries and LMICs [32]. While there is no systematic and comprehensive analysis of these other sources for human resources for health financing yet, understanding their landscape and comparing with the formal development assistance is of increasing importance and relevance in the global health labor market and should be addressed in future studies.

This study has several limitations. First the keyword search approach restricts relevance of projects to the descriptions provided in project documents so that if the implementation of a project deviates from what is provided in the description, we are unable to capture that change. This is the case with isolating projects pertaining to health workforce information systems in this study. From press releases, we can identify a handful of donors supported projects that include health workforce information system activities; however, those were not identified in the database. Even with these constraints, we believe the keyword search strategy is the best approach currently available while acknowledging that it is not perfect. Second, due to data limitation with some disbursing channel we were unable to further disaggregate by source or type, for example for some disbursing entities like Gavi and UN agencies we were only able to include in aggregate and did not have additional disaggregation by source in the analyses. For such disbursing agencies, we designated their source as unallocable. While the approach adopted ensures that the contributions through these agencies are accounted for, we acknowledge that for instance it also leaves about 54% (1.2 billion) of the support ($2.3 billion) in 2018 unaccounted for in terms of originating source. As such the estimates present a conservative estimate of the donor resources targeted towards health workforce-related activities; for others like China, while we can report China’s contribution to human resources for health in aggregate, we are unable to disaggregate it further according to type because data on that level of granularity is not publicly available. Third, because of the lag in data reported in the CRS we generated an estimate for 2020. In order to generate the estimate of donor funding for human resources for health-related activities in 2020, we leveraged the estimated development assistance for human resources for health envelope from IHME’s Development Assistance for Health data and used the weighted fractions of various types of activities supported in the previous 3 years to disaggregate the envelope by type. Therefore, the 2020 distribution of health workforce assistance may be different when actual data become available. We also do not estimate the proportion of the total DAH targeted towards gender-related activities in 2020 because of the same data limitation described above. Fourth, due to data limitation we were unable to disaggregate our estimate by different cadres and professional categories of the health workforce (community health workers, physician, nurses, etc.). Understanding investment by different cadres and linking them with country workforce data from national health workforce accounts and WHO/ILO/OECD inter-agency data exchange mechanism is important to direct resources to where they are most needed and to ensure a good balance of investment across cadres [33]. Lastly, our identification of gender-related projects relied on the gender marker variable included in the CRS database. While it is a credible marker because it is determined by the donors themselves who understand what their projects did, there is some evidence that suggests that there are differences in how different donors assign their projects. We also do not have gender estimates any donor for whom we did not use the CRS data. Our estimate is therefore likely a conservative minimum of the gender-related health workforce activities.

## Conclusions

Presently, the global health community is faced with multiple goals—global health security, universal health coverage and the sustainable development goals [34–36]—for which adequate availability and appropriate skill mix and distribution of health workers is critical to achieve. Since 2016, donor support for health workforce-related activities has increased. However, in the context of the primary role health workers play and what is needed for the attainment of the health-related Sustainable Development Goals, even more resources are needed and must be mobilized. Furthermore, there are lingering concerns related to the short-term nature of activities that donor funding supports and its viability for creating sustainable health systems. While it is important that short-term needs are addressed, it is also critical that development assistance for human resources also target the full range of activities that are necessary for deployment, retention and management of a sustainable skilled health workforce that can deliver on the global health goals. Milestone four of the Global Strategy on Human Resources for Health pushes for more synergy across donors. It is critical that both national governments and development partners are engaged in advancing a deliberate strategy that actively manages planned investments for long-term sustainability of the health sector.

## Supplementary Information

**Additional file 1**: Supplementary methods Annex. Development assistance for the Global Strategy for Human Resources for Health; 1990-2020. Version: December 22, 2021. **Table S1.1. **Terms for keyword searches.** Table S1.2. **Types of activities—terms for keyword searches.

## Data Availability

The datasets generated and/or analyzed during the current study are available in the Global Health Data Exchange database https://ghdx.healthdata.org/record/ihme-data/development-assistance-health-database-1990-2020.

## Abbreviations

DAH: Development Assistance for Health
GFATM: The Global Fund to Fight AIDS, Tuberculosis and Malaria
GSHRH: Global Strategy on Human Resources for Health
IHME: Institute for Health Metrics and Evaluation
OECD: Organisation for Economic Cooperation and Development
OECD CRS: Organisation for Economic Cooperation and Development Creditor Reporting System
PEPFAR: The U.S. President’s Emergency Plan for AIDS Relief
UN: United Nations
WHO: World Health Organization
