# Supplementary material for: Development assistance for human resources for health, 1990–2020
Source: Hum Resour Health. 2022 Jun 10;20:51. doi: 10.1186/s12960-022-00744-x (PMC9187148; doi:10.1186/s12960-022-00744-x)
Supplement: Supplementary file 1 — Additional file 1: Supplementary methods Annex. Development assistance for the Global Strategy for Human Resources for Health; 1990-2020. Version: December 22, 2021. Table S1.1. Terms for keyword searches. Table S1.2. Types of activities—terms for keyword searches. [file 12960_2022_744_MOESM1_ESM.docx]

SUPPLEMENTARY METHODS ANNEX

## Development assistance for the Global Strategy for Human Resources for Health; 1990-2020

Version: December 22, 2021

**Table S1.1.** **Terms for keyword searches**

| Health focus area level | Program area | Keywords |
| --- | --- | --- |
| Human Resources for Health | HRH | " INFRASTRUCTUR" " MEDICAL EQUIPMENT" " SURGICAL EQUIPMENT" " HOSPITAL EQUIPMENT" " HOSPITAL EQMT " " BUILDINGS " " HEALTH FACILIT" " CONSTRUCT" " MEDICAL SCHOOL" "CENTERS OF EXCELLENCE" " TRAINING " " CAPACITY " " SKILLED WORKER" " HEALTH WORKER" " SKILLED STAFF " " HEALTH PROFESSIONAL" " HUMAN RESOURCE" " HUMAN CAPITAL " " IMPROVED CAPACITIES " " MEDICAL WORKER" " HEALTH CARE PERSONNEL " " WORKFORCE " " ADMINISTRATIVE " " MEDICAL EDUCATION " " HEALTH EDUCATION " " CONTINUING EDUCATION " " HEALTH MANAGEMENT" " MANAGEMENT AND COORDINATION " " ADMINISTRATIVE MANAGEMENT " " MANAGEMENT AND ADMINISTRATION " " STRENGTHENING INSTITUTIONAL CAPACIT" "NURSE" "DOCTOR" "PHYSICIAN" "MIDWIFE" "MIDWIVES" "MEDICAL LABORATORY SCIENTIST" "SURGEON" "SPECIALIST" "PHARMACIST" "COMMUNITY HEALTH WORKER" "EMPLOYMENT" "HEALTH LABOR" "LABOR MARKET" "PERSONNEL" "MEDICAL PRACTIONER" "DENTAL PRACTIONER" "TASK SHIFTING" " ALLIED HEALTH WORKERS " " TASK SHARING " " TASK SHIFTING " " COMPETENCIES " " HEALTH WORKFORCE DEVELOPMENT " " STRATEGIC PLAN " " HEALTH WORKFORCE POLICIES " " INFORMATION " " INFORMATION SYSTEM " " GENDER - WOMEN " |

**Table S1.2.** **Types of activities** - **Terms for keyword searches -**

| Program area | Keywords |
| --- | --- |
| Training | "TRAINING" " TRAIN " "TRNG" "CAPACITY" "CAPACITIES" "CAPACITE" "ENTRAINEMENT" "LEARNING" "INTERNSHIP" " FORMATION" "WORKSHOP" "COURSES" |
| Education | " EDUC" "SCHOLARSHIP" "BOURSES" |
| Admin and Policy | " ADMIN" "MANAGEMENT" "LEADERSHIP" "POLICY" "POLICIES" |
| Personnel | "PERSONNEL DEVELOPMENT" "DVPT" "DEVPT" |
| Staffing | "FELLOW" "SALAR" "VOLUNTEER" "STAFFING" "PROVISION OF SURGICAL TEAM" "CONSULTANCY" "SECONDMENT" "WORK PERMIT" "TA SUPPORT" "TECHNICAL ASSISTANCE" |
| General support | "GENERAL BUDGET SUPPORT" |
| Infrastructure | " INFRASTRUCTUR" " MEDICAL EQUIPMENT" " SURGICAL EQUIPMENT" " HOSPITAL EQUIPMENT" " HOSPITAL EQMT " " BUILDINGS " " HEALTH FACILIT" " CONSTRUCT" " MEDICAL SCHOOL" "CENTERS OF EXCELLENCE" |
| Health workforce information systems | " HEALTH WORKFORCE INFORMATION SYSTEM" " HUMAN RESOURCES FOR HEALTH INFORMATION SYSTEM" " HRHIS " " HEALTH CARE WORKER AND TRACKING " " HRH PROFILE" " HUMAN RESOURCE INFORMATION SYSTEM" |
